# Attenuation From Shoes and Pads in Equine Nuclear Scintigraphy, With Relevance to Solar Views

**DOI:** 10.3389/fvets.2020.516718

**Published:** 2020-09-24

**Authors:** Lea Walker, Mark J. Martinelli, Norman Rantanen, Bianca Drumond, Steven Trostle

**Affiliations:** ^1^California Equine Orthopedics, San Marcos, CA, United States; ^2^Blue Ridge Equine Clinic, Earlysville, VA, United States

**Keywords:** equine, scintigraphy, attenuation, shoe, Pad, solar view

## Abstract

Nuclear scintigraphy can be beneficial in the investigation of equine lameness and poor performance. Images obtained through the sole allow for better identification of a region of increased radionuclide uptake in the foot. The presence of shoes or pads may affect these images. To determine the degree of radioactive attenuation different types of shoes and pads of different thickness and materials were evaluated by placing the material directly on the gamma camera detector acquiring flood images from a point source. The study compared five different types of shoes from 3 different shoeing materials, steel, aluminum, and polyurethane. This study also assessed 8 different types of pads that were selected based on the subjective densities and variable rigidity of the materials. All types of shoes/pads evaluated caused some degree of attenuation (*P* < 0.05). Steel shoes of all types cause the most attenuation (54%), followed by aluminum shoes (22%), and the shoes or pads composed primarily of a plastic polymer cause the least amount of attenuation (15%). The results of the study found that pads or shoes cause significant (*p* < 0.05) attenuation of gamma radiation. Composition, thickness and density characterized mass absorption coefficient, of the material affect the amount of gamma radiation attenuation. Because of the attenuation of gamma radiation, we recommend shoes and pads be removed prior to nuclear scintigraphic examination of the front foot if a solar image is desired, as this attenuation may affect image quality.

## Introduction

Nuclear scintigraphy of the equine foot is an established imaging modality in the investigation of equine lameness and poor performance (1). Increased radiopharmaceutical uptake (IRU) has been described by researchers and clinicians in association with abnormalities of the navicular bone (1–5) collateral ligaments of the distal interphalangeal joint (2, 3, 6), deep digital flexor tendon (1, 2), distal phalanx (2, 7, 8) and ungular cartilages (9–11) and used to evaluate laminar perfusion (12). Nuclear scintigraphy of the equine musculoskeletal system has been considered to be very sensitive, but not specific for injuries in lame or poorly performing horses (1, 2). Although MRI, and to a lesser degree CT, are the predominant techniques for advanced imaging of the foot, nuclear scintigraphy can provide additional clinically useful information. The value of nuclear scintigraphy has been documented for several anatomic sites within the foot (1, 3). The agreement between both increased radiopharmaceutical uptake and relevance categorization with the presence of foot pain is substantial (13), and a lack of radiopharmaceutical uptake does not preclude injury and can still provide potentially important information in the foot (7).

For assessment of uptake of the structures in the foot, lateral/medial, dorsal/palmar and solar scintigraphic views are necessary (3, 9, 14). Different types of shoes or shoe/pad combinations may reduce the ability of the practitioner to clearly identify the different structures, making accurate diagnosis more difficult, especially on the solar views. There is limited information correlating types of shoes and pads with the amount of attenuation of the radiation. It has been recommended in some circumstances, particularly with eggbar and heartbar shoes that may effectively act as a mask or normal open shoes that shield parts of the bones of the foot, to remove the shoes to obtain an adequate solar image (15).

The purpose of this study was to quantify the percent attenuation of gamma radiation by different types and materials of shoes and pads commonly worn by horses undergoing nuclear scintigraphic imaging. It is hypothesized that different types and materials of shoes and pads will cause attenuation of the solar view. Attenuation of gamma radiation effectively increases the noise in the data received by the detector, even if the attenuation is uniform across the solar view. Therefore, we propose in a clinical setting that in the presence of a shoe or pad some information will be inadvertently masked or lost, which may impact the ability of the clinician to accurately interpret a solar image.

## Materials and Methods

### Imaging, Instrument Calibration, and Uniformity

Images were acquired on a NuCam gamma camera (Anger Type) mounted on an EqView stand both produced by Diagnostic Services Inc. (Middlesex, NJ) and interfaced to a Mirage Imaging Computer produced by Segami Corp (Columbia, MD). The camera includes a 0.95-cm thick NaI (sodium iodide) crystal optically interfaced to 55 photomultiplier tubes. Energy level and X-Y position of gamma events are determined at the detector. The Mirage computer digitizes the signal, applies isotope selection, energy window selection and applies energy, linearity, and uniformity corrections. Images were acquired in a 256 by 256 matrix on a 51-cm by 37-cm field of view. Flood images were generated using a calibration dose (37 Mbq/1.0 mCi) of radioactive technetium-99 (^99m^Tc) as sodium pertechnetate, placed in small tube and located several meters away from the camera. Radioactive ^99m^Tc emits a 140 keV gamma ray with a half-life of 6.0 h. NEMA (National Electrical Manufacturers Association) internal uniformity tests were performed and passed for use of the instrument. The NuCam gamma camera assembly includes a lead collimator to enhance image focus and resolution. The collimator was removed to ensure that a uniform radiation background is obtained and to reduce the data acquisition times.

### Control and Attenuation Data Acquisition

Internal calibration and uniformity testing of the instrument was completed. Randomized multiple sets of shoes or pads were placed directly on the surface of the camera and flood images of the field were acquired to total 3,000,000 counts. A total of four “sets” of shoe and/or pad images were obtained, each set consisting of a total of as many as four shoes or pads per camera view. Data was acquired for each set three times to reduce imaging error. The three acquisitions were compared to each other to confirm reproducibility and field uniformity. One set was acquired four times. The fourth image of that set was acquired for a total of 12,000,000 counts to accurately trace the margins of the pads in that image. Additionally, the higher counts also improve the statistical uncertainties for the pads. The acquired images are in Appendix 1.

A summary of the materials used in each of the four sets of data is summarized in Table 1. In each image acquired, measurements of identical areas (ROIs) in three unobstructed “control” locations on the field (measured in points through the instrument's Mirage software, and points are analogous to pixels on a computer screen) were taken to determine the number of counts in a given area (counts per point). The “control” incident radiation detected as counts/point is designated *I*_0_.

**Table 1:** Sets of Shoes/Pads Imaged Concurrently.

**Set 1**	**Set 2**	**Set 3**	**Set 4**
Standard steel shoe	Green impact pad	Aluminum spider plate	Small aluminum wedge shoe
Steel heartbar shoe	Black impact pad	Black spider plate	Large aluminum standard shoe
Steel eggbar shoe	Leather impact pad	Waffle impact pad	Plastic shoe
Small aluminum wedge shoe	Clear wedge impact pad	Plastic shoe	Green rim pad

Additionally, for each data set, the outline of each shoe or pad was traced creating a ROI. The area and the counts for each ROI were generated (*I*, as counts per point). Due to the variable thickness of the pads and some shoes, two types of ROI (*I*) measurements were performed. Small, uniform circular areas in multiple locations were measured and also, the entirety of the pad or shoe was traced.

The average of these control measurements within and across acquisitions for a set was used to evaluate the degree of attenuation of a given shoe or pad ROI. The counts per point for each shoe or pad ROI (I) in each data set was compared to the average control counts per point in each data set and percent attenuation was generated (16).

(1) $$\begin{array}{l} \text{Attenuation}=A=\left[1-I\left(shoe/pad\right)/I_{0}\left(control\right)\right]*100 \end{array}$$

These data and results of the attenuation calculations are presented in the Results (section Results). In the data sets, estimates of *I*_0_ use small circular ROIs traced over portions of the image where no obstructing materials exist. For example, in Set 1 there are five control ROIs used over a total of three acquisitions of data. In the first acquisition, there are three control ROIs located (a) at the upper left, (b) at the image center, and (c) at the bottom center. Acquisitions 2 and 3 have a control ROI located at the center of the images identical in size and location to the central ROI in acquisition 1. These 5 ROIs were used to confirm the spatial field uniformity across the camera face in a single acquisition and the temporal field uniformity at a single spot in the center of each image obtained in three successive acquisitions.

### Effect of Shoes and Pads

The study compared five different types of shoes (standard aluminum (2 sizes), standard steel, steel eggbar, steel heartbar, and plastic (Epona, Epona Shoe, Inc., Creston, CA 93432 USA) made from 3 different shoeing materials. This study also assessed several different types of pads. A black plastic impact pad, a green plastic impact pad, a clear plastic wedge pad with frog support, a black plastic spider plate, a green rim pad, an aluminum spider plate, a leather impact pad and a “waffle-pad” were all evaluated. The different plastic materials were chosen based on the subjective densities and variable rigidity of the materials. The clear plastic material was hardest and most rigid of them, whereas the waffle-pad was the softest and most pliable. The green plastic impact pad and rim pad were slightly more rigid than the waffle pad and the black plastic materials (impact pad and spider plate) were slightly less rigid than the clear plastic pad. The thicknesses of the shoes and pads are listed in Table 2.

**Table 2:** Shoe and Pad Materials and Thicknesses.

	**Material**	**Thickness**	
**Shoes**			
Standard	Steel	7.5 mm	
Standard	Aluminum	Heel- 13.5 mm	Toe- 9.5 mm
Heartbar	Steel	9.0 mm	
Eggbar	Steel	8.0 mm	
Small wedge	Aluminum	Heel- 13.9 mm	Toe- 8.4 mm
Plastic	Polyurethane	14.5 mm	
**Pads**			
Impact pad	Soft green plastic wedge	Heel- 7.3 mm	Toe- 1.3 mm
Impact pad	Hard black plastic	5.0 mm	
Impact pad	Leather	5.3 mm	
Impact pad	Hard clear wedge	Heel- 11.3 mm	Toe- 1.8 mm
	with frog support	Frog- 12.0 mm	Frog center- 4.3 mm
Impact pad	Soft clear “waffle” plastic	5.3 mm	
Impact pad	Green rim	5.0 mm	
Spider plate	Aluminum	3.0 mm	
Spider plate	Black plastic	5.5 mm	

### Statistical Analysis of Background Measurements, Shoe and Pad Attenuation Data

Statistical analysis of background ROIs and of the shoe and pad ROI data was performed using ANOVA calculations through a web-based interface at (URL: http://statpages.info/anova1sm.html, retrieved 10/15/2018), using total gamma ray counts, count rate means, and standard errors of the means (the uncertainties in the count rates alone). The standard errors of the mean decrease with larger counts and larger ROIs (number of points).

Descriptive statistical means and standard deviations of the means were calculated and summarize the percentage of attenuation by different shoes and pads. Comparison of gamma radiation attenuation measurements was performed using repeated ANOVA calculations. For all analysis, *p* < 0.05 were considered statistically significant.

## Results

### Summary Data for Background Measurements

Data for unobstructed regions of interest (*I*_0_) can be found in Table 3. Control measurements for each data set (*I*_0_) confirm the uniformity of the field and normalize for radiopharmaceutical decay during the procedure. Counts and points in the table are raw data taken from information printed on the generated images using the Mirage Imaging software.

**Table 3:** Counts per point in unobstructed regions of interest in each of 4 image sets.

**Set #**	**Location on image**	**Image in set**	**Counts**	**Points (pixels)**	**Counts/point**
Set 1	ROI 0	Image 1	10,964	208	52.71
Set 1	ROI 1	Image 1	11,011	209	52.68
Set 1	ROI 2	Image 1	11,097	209	53.10
Set 1	ROI 0	Image 2	11,023	208	53.00
Set 1	ROI 0	Image 3	10,946	208	52.63
**Set 1**	**Average I**_**o**_				**52.82**
Set 2	ROI 0	Image 1	10,502	208	50.49
Set 2	ROI 1	Image 1	10,720	210	51.05
Set 2	ROI 2	Image 1	10,857	209	51.95
Set 2	ROI 0	Image 2	10,579	208	50.86
Set 2	ROI 0	Image 3	10,548	208	50.71
**Set 2**	**Average I**_**o**_				**51.01**
Set 2^*^	ROI 0	Image 4^*^	42,268	208	203.21
**Set 2**	**I**_**o**_	**Image 4**			**203.21**
Set 3	ROI 0	Image 1	10,508	208	50.52
Set 3	ROI 1	Image 1	10,626	209	50.84
Set 3	ROI 2	Image 1	10,492	210	49.96
Set 3	ROI 0	Image 2	10,476	208	50.37
Set 3	ROI 0	Image 3	10,465	208	50.31
**Set 3**	**Average I**_**o**_				**50.40**
Set 4	ROI 0	Image 1	10,648	208	51.19
Set 4	ROI 1	Image 1	10,813	208	51.99
Set 4	ROI 2	Image 1	10,747	209	51.42
Set 4	ROI 0	Image 2	10,615	208	51.03
Set 4	ROI 0	Image 3	10,472	208	50.35
**Set 4**	**Average I**_**o**_				**51.20**

*Bolded values are the averages from each set*.

#### Statistical Analysis of Background Measurements

An ANOVA was used to calculate the means and standard deviations of ROIs 0, 1, and 2 from acquisition 1 of Set 1 (Table 3). The corresponding ANOVA calculation returns a *p*-value of *p* = 0.80, indicating the amount of variation in the count rates for these three ROIs would be expected ~80% of the time if the samples are taken from the same underlying distribution. Secondly, if each field is uniform between two different acquisitions made for a given set of data, then it will not matter if the control ROI is extracted from one acquisition and the shoe/pad ROI is extracted from a subsequent or prior acquisition. ANOVA was used to compare the means and standard deviations of ROI 0 from acquisitions 1, 2, and 3 of the Set 1 data, also in Table 3. This ANOVA calculation returns a *p*-value of *p* = 0.86, indicating we can expect to see this much variation between means ~86% of the time. The background measurements and analysis for each set (*I*_0_) in Table 3 confirm the uniformity of the field and from one acquisition to the next. This process also normalizes for radiopharmaceutical decay during the procedure since it is concurrent with the ROI measurements of the shoes and pads.

The measured background, used to calculate values of *I*_0_ is statistically uniform, both within a single acquisition image and from one acquisition to the next.

### Summary Data for Shoes and Pads

**Figure 1:**
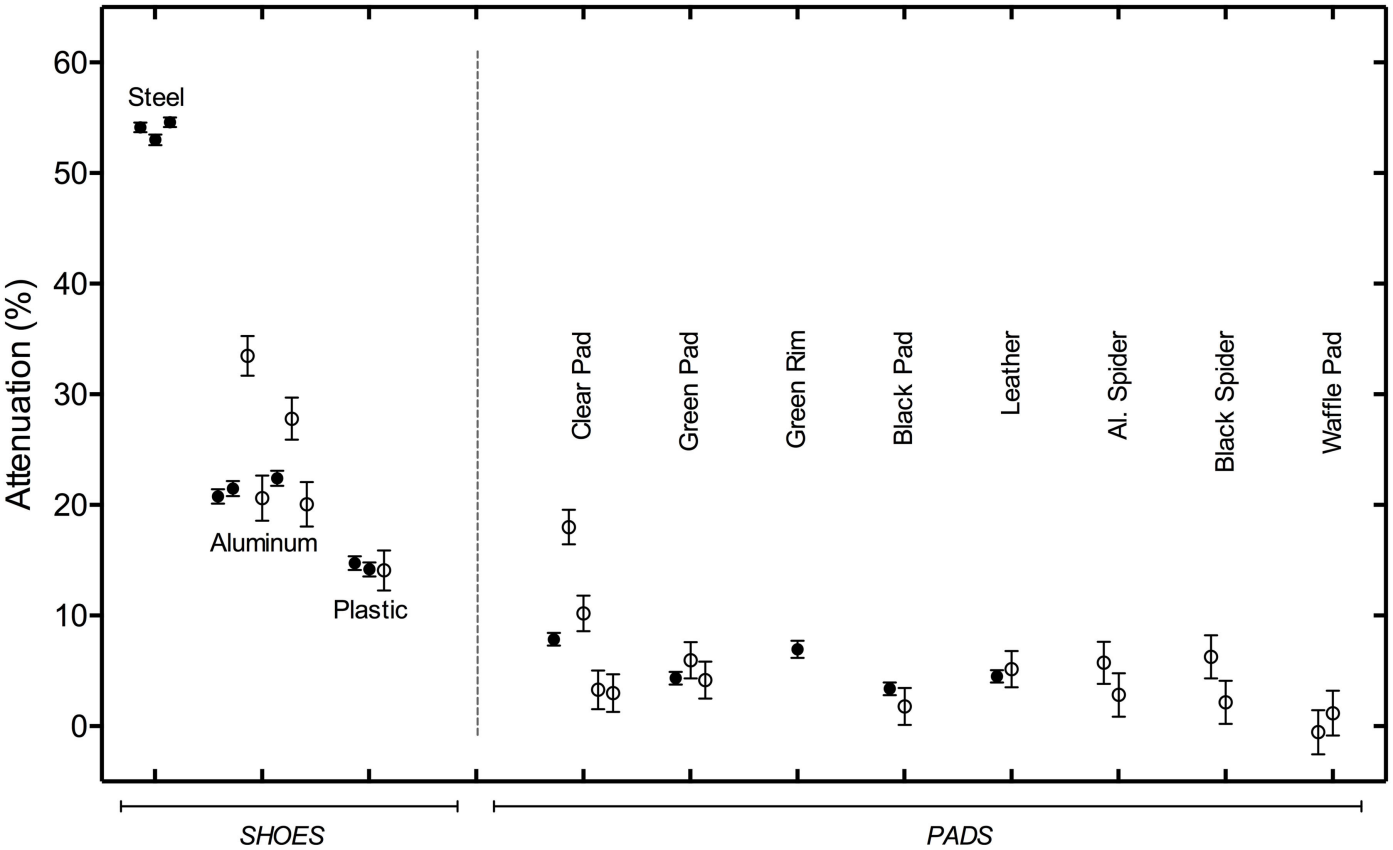
Shoe and Pad Attenuation (with uncertainties). Attenuation for each shoe or pad imaged is shown as a circle for the mean attenuation, with error bars for the 95% confidence level. Filled circles indicate that the entire shoe or pad was traced, and open circles indicate ROI where a localized spot trace was used. Error bars on the spot traces are generally larger because of the smaller area included in the ROI. Two shoes were imaged twice, in different data sets. The difference shown for the two measurements of the attenuation of the small aluminum shoe and the plastic shoe shows how reproducibly the shoe outline could be traced.

Data for shoes/pads and calculated attenuation (1-*I/Io*)100 % using equation 1 is presented in Tables 4, 5 and a summary of the observed attenuation data is presented graphically in Figure 1. Counts and points in the tables are raw data taken from information printed on the generated images using the Mirage Imaging software. The corresponding average *I*_0_ for each set is found in Table 3. An expression for the uncertainties associated with the measurements was derived using calculus of variations, so that

(2) $$\begin{array}{l} \delta \;\left(A\right)=100\;\alpha \frac{C_{M}^{1/2}}{C_{B}}\left(1+\frac{C_{M}^{1/2}}{C_{B}^{1/2}}\right) \end{array}$$

where α is the ratio of background counts to counts when obstructing materials are present, and *C*_*M*_ and *C*_*B*_ are the total counts in a region of interest that contains materials (*C*_*M*_) or only contains background counts (*C*_*B*_).

**Table 4:** Data for Shoes (I) and Attenuation Calculation.

**Set #/image#/location**	**ROI type**	**Material description thickness (mm)**	**Counts**	**Points (pixels)**	**Counts/point (I)**	**(1-I/I_**o**_)100% percent attenuation ± uncertainty calc. from equation 2**
**Shoes**						
Set 1/image 3/ROI 0	Full trace	Steel- heartbar 9.0 mm	41,065	1,695	24.23	54.13 ± 0.42^a^
Set 1/image 3/ROI 1	Full trace	Steel – standard 7.5 mm	26,239	1,057	24.82	53.00 ± 0.49^a^
Set 1/image 3/ROI 2	Full trace	Steel- eggbar 3.0 mm	33,987	1,417	23.99	54.59 ± 0.44^a^
Set 1/image 3/ROI 3	Full trace	Aluminum- small 8.4–13.9 mm	61,562	1,471	41.85	20.77 ± 0.66^b^
Set 4/image 3/ROI 3	Full trace	Aluminum- small 8.4–13.9 mm	55,639	1,384	40.20	21.48 ± 0.67^b^
Set 4/image 3/ROI 1a^β^	Spot area	Aluminum small heel (13.9 mm)	1,941	57	34.05	33.49 ± 1.^80b^
Set 4/image 3/ROI 3a^β^	Spot area	Aluminum small toe (8.4 mm)	2195	54	40.65	20.60 ± 2.04^b^
Set 4/image 3/ROI 1	Full trace	Aluminum large 9.5–13.5 mm	52,515	1,322	39.72	22.41 ± 0.67^b^
Set 4/image 3/ROI 0a^β^	Spot area	Aluminum large heel (13.5 mm)	2,070	56	36.96	27.80 ± 1.90^b^
Set 4/Image 3/ROI 2a^β^	Spot area	Aluminum large toe (9.5 mm)	2,292	56	40.93	20.06 ± 2.02^b^
Set 4/image 3/ROI 0	Full trace	Plastic 14.5 mm	114,739	2,629	43.64	14.75 ± 0.62^c^
Set 3/image 3/ROI 0	Full trace	Plastic 14.5 mm	112,725	2,606	43.26	14.17 ± 0.63^c^
Set 3/image 3/ROI 7	Spot area	Plastic center 14.5 mm	3,551	82	43.30	14.08 ± 1.82^c^

β *Where a second copy of an image was used for additional ROIs, a suffix designation “a” was added to indicate those ROIs*.

a, b, c *Within a column, means without a common superscript differ (p < 0.05); meaning the attenuation caused by steel shoes (a) was statistically different from that caused by aluminum shoes (b) and both are statistically different from that caused by Plastic shoe (c)*.

**Table 5:** Data for Pads/Plates (I) and Attenuation Calculation.

**Set #/image#/location**	**ROI type**	**Material description thickness (mm)**	**Counts**	**Points (pixels)**	**Counts/point (I)**	**(1-I/I_**o**_)100% Percent attenuation ± uncertainty calc. from equation 2**
**Pads/plates**						
Set 2^α^/image 4/ROI 2	Full trace	Impact pad Clear hard 1.8–11.3 mm	671,066	3,584	187.24	7.86 ± 0.56^a^
Set 2^α^/image 4/ROI 2a^β^	Spot area	Impact pad Clear hard Heel clear frog 12.0 mm	4,999	30	166.63	18.00 ± 1.56^b^
Set 2^α^/image 4/ROI 7a^β^	Spot area	Heel clear wing 11.3 mm	5,839	32	182.47	10.21 ± 1.61^a^
Set 2^α^/image 4/ROI 0a^β^	Spot area	Center clear frog 4.3 mm	5,699	29	196.52	3.29 ± 1.75^a^
Set 2^α^/image 4/ROI 6a^β^	Spot area	Toe clear frog 1.8 mm	6,111	31	197.13	2.99 ± 1.71^a^
Set 2^α^ /image 4/ROI 0	Full trace	Impact pad Green 1.3–7.3 mm	909,363	4,678	194.39	4.34 ± 0.57^a^
Set 2^α^/image 4/ROI 1a^β^	Spot area	Green heel 7.3	6,307	33	191.12	5.95 ± 1.64^a^
Set 2^α^/image 4/ROI 5a^β^	Spot area	Green toe 1.3 mm	6,427	33	194.76	4.16 ± 1.66^a^
Set 4/image 3/ROI 2	Full trace	Impact pad Green rim	62,795	1,318	47.64	6.94 ± 0.77^a^
Set 2^α^/image 4/ROI 1	Full trace	Impact pad Black 5.0 mm	947,891	4,828	196.33	3.39 ± 0.57^a^
Set 2^α^/image 4/ROI 3a^β^	Spot area	Black center Not measured	6,786	34	199.59	1.78 ± 1.67^a^
Set 2^α^ /image 4/ROI 3	Full trace	Impact pad Leather 5.3 mm	852,635	4,394	194.05	4.51 ±0.57^a^
Set 2^α^/image 4/ROI 4a^β^	Spot area	Leather center 5.3 mm	6,360	33	192.73	5.16 ± 1.65^a^
Set 3/image 3/ROI 1	Spot area	Aluminum spider Rim 3.0 mm	3,991	84	47.51	5.73 ± 1.90^a^
Set 3/image 3/ROI 2	Spot area	Aluminum spider Center Not measured	4,016	82	48.98	2.83 ± 1.96^a^
Set 3/image 3/ROI 3	Spot area	Black spider Rim 5.5 mm	3,685	78	47.24	6.26 ± 1.95^a^
Set 3/image 3/ROI 5	Spot area	Black spider Center Not measured	4,093	83	49.31	2.16 ± 1.96^a^
Set 3/image 3/ROI 4	Spot area	Waffle impact Heel 5.3 mm	4,155	82	50.67	−0.54 ± 2.00^a^
Set 3/image 3/ROI 6	Spot area	Waffle impact Toe 5.3 mm	3,835	77	49.81	1.18 ± 2.03^a^

α *Percent Attenuation and regions of interest (ROIs)were calculated using Image 4 in Set 2 that was acquired for 12,000,000 counts to improve the observer's ability to correctly identify the margins of the pads*.

β *Where a second copy of an image was used for additional ROIs, a suffix designation “a” was added to indicate those ROIs*.

a *The attenuation caused by these pads was not statistically differently from one another*.

b *The attenuation caused by the clear impact pad at the heel over the frog was statistically different from the attenuation caused by the rest of the pads (P < 0.05)*.

The uncertainties reported in Tables 4, 5 depend on the total number of counts accumulated in the regions of interest. The uncertainty in the number of counts follows from the fact that gamma ray detection follows a Poisson distribution (16), so that the corresponding uncertainty in counts for a ROI is the square root of the total counts. All statistical analyses were performed by Robert B. Walker, PhD and a complete derivation of this expression is available by request to the first author.

#### Statistical Analysis of Shoe and Pad Attenuation Data

ANOVA confirms that although the center and rim attenuation measurements of the aluminum spider plate have slightly different attenuations (2.8 vs. 5.7%), they are not statistically different (*p* = 0.17) from each other. For the Large Aluminum Standard shoe, the heel and toe attenuations are 27.8 and 20.1%, respectively, corresponding to maximum thickness measurements of 13.5 and 9.5 mm (although average effective thicknesses are smaller, due to irregularities in the shoe). ANOVA calculations demonstrate that these attenuation differences are statistically significant, *p* = 0.0008, in part because the attenuations are higher as a whole. The overall attenuation for the Large Aluminum Shoe was 22.4%, closer in value to the toe measurement since this shoe has a thicker heel. An ANOVA calculation comparing the whole shoe, and the toe and rim, shows that the count rates for the heel are significantly different than for the overall shoe or the toe (*p* = 0.0022 and 0.0044), but the shoe and toe count rates are not significantly different (at *p* = 0.31).

Differences in count rates can occur within a single pad. The clear pad shows definite structure in the acquired image data, with the toe producing the least attenuation (2.99%) and the frog heel portion producing the most (18.0%), with associated maximum thickness measurements of 1.8 and 12.0 mm. ANOVA calculations show the spot ROI count rates to be statistically different at *p* = 0.029.

When comparing classes of shoes (Steel vs. Aluminum vs. Plastic), using the combined counts and ROIs for the three steel shoes, the two aluminum shoes, and the plastic shoe, all three sets of attenuations are statistically significant (with *p* < 0.0001).

## Discussion

All shoes regardless of type or material caused some degree of attenuation of gamma radiation. As a group, shoes caused noticeably more gamma radiation attenuation than the pads. Regardless of the shoe type, shoes made of steel (standard, eggbar and heart bar) provided the greatest average attenuation (53.9%), followed by aluminum shoes (21.59%) and then plastic shoes (14.75%). All pads caused some degree of attenuation of gamma radiation. All the pads had <10% average attenuation. The thickness of the pad and shoe material is the most important factor, aside from the material, in the amount of radiation that was attenuated, as evidenced the variable thickness of the clear plastic impact pad causing more attenuation at the thickest parts of the pad. But pads in general are thinner than shoes, typically cover the entire solar view, and so yield small overall attenuations. This is graphically represented in Figure 1.

At our γ-ray energy of 140 keV, the mass absorption coefficient is the sum of the partial mass absorption coefficients from the photoelectric and Compton scattering effects. As this physics has been well-described, tables of mass absorption coefficients in cm^2^/g are readily accessible in the literature (17). The mass absorption coefficient of Compton scattering decreases with Z number while the photoelectric effect increases with Z number. The elements of interest are iron-Z = 26 (steel), aluminum-Z = 13 and carbon-Z = 6 (polymer such as polyurethane) since these are the elements that are of the highest concentration in the materials studied here. The total μ*(mass absorption)* coefficients, including both photoelectric and Compton scattering effects at near 140 keV for iron, aluminum and carbon are 0.184 (0.1240 + 0.0595), 0.134 (0.1288 + 0.0052), and 0.134 (0.1336 + 0.0003) cm^2^/g, respectively, where the numbers in parentheses are Compton scattering + photoelectric effect for each element.

To demonstrate the relationship of thickness to attenuation, Table 6 shows how predicted attenuations vary with thickness for each of the three major shoe materials in this study. The three parameters that define the predicted attenuation are the mass absorption coefficient, density, and thickness of the attenuating material. The first parameter depends on the element and the gamma ray photon energy previously discussed. For the second parameter we used common densities for steel and elemental aluminum but polymers may have a variety of densities depending on the manufacturing process (the error in the average density for Epona polyurethane could be different from the polymer in the table). A mid-range density of 1.2 g/cm^3^ for polyurethane is used here. The third parameter is thickness but may not be as simple as a single measurement. Most shoes have an irregular surface with shaping, grooves, and other structural features. Measurements are only of the thickest part of the profile and will lead to overestimated attenuation if raw data from Table 2 is used. For these reasons one would not expect the predicted attenuations to exactly match the observed attenuations.

**Table 6:** Prediction of Attenuation for Different Materials and Thicknesses at 150 keV.

**Material**	**mass abs coeff(mac)**	**density(d) g/cm^**3**^**	**representative**	Transmitted fraction e^(−d^*^mac*x)^	**predicted% attenuated**	**observed% attenuated**
			**thickness(x) cm**			
**Steel**						
	0.184	8	0.50	0.479	52.1	54
			0.75	0.332	66.8	
			1.00	0.229	77.1	
**Al**						
	0.134	2.7	0.60	0.805	19.5	21
			0.80	0.749	25.1	
			1.00	0.696	30.4	
**Polymer**						
	0.134	1.2	1.00	0.851	14.9	14
			1.50	0.786	21.4	

To adequately appreciate the potential impact that the shoe and pad materials may have on nuclear scintigraphic images, it is important to understand the basic physics of gamma radiation and how images are generated.

The chemical and physical interaction of gamma-rays (γ-rays) with matter has been well-described in the literature (16–18). Gamma radiation is absorbed in matter differently than absorption that occurs with charged particles. Its interaction is primarily with atomic electrons and the γ-ray can lose a large fraction, or all, of its energy in a single encounter. The rays are absorbed according to an exponential law, characterized by a half thickness and μ (linear absorption coefficient). The equation below provides the intensity (*I*) of the rays after the initial intensity (*I*_0_) of a beam of γ-rays has passed through *x* cm of absorbing matter.

(3) $$\begin{array}{l} I={Io\;e}^{-\mu \left(linear\right)x} \end{array}$$

However, published values of material absorption coefficients μ*(mass absorption)* are expressed in units of (cm^2^/g), and to use those values to calculate expected attenuation values, you must also use the material density D (g/cm^3^), to obtain the linear absorption coefficient μ as

(4) $$\begin{array}{l} \mu =\mu \left(linear\right)=D*\mu \left(mass\;absorption\right) \end{array}$$

We can combine equations to get:

(5) $$\begin{array}{l} \frac{I}{Io}=e^{-\mu \left(mass\;absorption\right)Dx} \end{array}$$

where *I/Io* is the fraction detected, *D* is the density of the material in g/cm^3^, *x* is the thickness in cm and the mass absorption coefficient is in cm^2^/g. Using density data for polyurethane, aluminum and steel, μ*(mass absorption)* and thicknesses measured from the materials in Table 2, we can predict attenuations of some materials in this study in Table 6. This validation of findings suggests that if a clinician knows the composition (density) and thickness of a given shoe or pad, a prediction of the percent attenuation can be made.

Compton scattering is the only source of extraneous radiation that could result from the shoe, because the photoelectric effect produces only electrons, not photons. However, in clinical practice typically two measures are used to prevent extraneous photons from being detected and interfering with the image generated from the source (i.e., the horse's foot). First, during image acquisition *in vivo*, the gamma camera is used with a lead collimator. The collimator narrows the beam of γ-rays, selecting only those that are aligned in a specific direction, namely, rays perpendicular to the camera sensor. Using the collimator in this study would have resulted in a non-uniform field and would have required much longer acquisition times (or a stronger source). Secondly, the camera software allows for “gating,” whereby the photomultiplier tubes only detect γ-rays within a narrow range of energies (135–141 keV). This “gating” prevents photons of a lesser energy, such as those generated by Compton scattering, from being detected and interfering with the image and is used in this study and in the clinical setting. The image acquisition methodology used in this study also differs from what is done in a clinical setting. For this study, two modifications were used to create a uniform flood of gamma rays over the field: (1) the collimator was removed, and (2) the Tc source was placed several meters away from the sensor to effectively create a point source. A flood of the field was necessary in this study, as it is the only way to ensure uniformity of the gamma radiation hitting the detector and allows us to eliminate any potential variables affecting the data from a non-uniform field. The point source is several meters from the detector and therefore the photons hitting the detector are essentially at 90-degrees to the plane of the detector and the shoe is placed directly on the detector so no photons could hit the camera from other angles making the collimator unnecessary and will greatly reduce the count time. These two modifications do not mimic a clinical acquisition of data; however they were necessary to get an accurate calculation of the percent of attenuation and will give an accurate measure of the attenuating properties of the shoes and pads. In a clinical setting, the uptake of gamma radiation of interest is generated from bone, whereas the measurement of interest in this particular study is the interaction of the gamma radiation with the material and what is detected by the camera. Additionally, it was necessary to eliminate unknown variables such as movement, poor bone uptake and undiagnosed underlying pathology, from the current study to allow for comparative data for *in vivo* studies.

We propose that without removal of the shoe, the ability of the clinician to accurately identify the structure or structures associated with IRU on the solar view may be inhibited by attenuation of gamma radiation of clinically important structures and removing the shoe may improve the clinician's ability to make an accurate assessment of all the regions of interest. Additionally, we propose, when shod with a pad, there is further attenuation of the gamma radiation, which can further inhibit an accurate read of the solar image. Although plastic shoes caused significantly less attenuation than steel or aluminum shoes the attenuation is still considered statistically significant. Additionally, plastic shoes may not be a uniform thickness and often do not cover the entire sole, which may make clinical image interpretation in the presence of a plastic shoe difficult outside of just the percent attenuation caused by plastics of a uniform thickness. This concept is also illustrated by the measurements of clear plastic impact pad. The goal of this study was to attempt to quantify the amount of attenuation a given shoe or pad creates. Although attenuation is cumulative, it is not additive, because individual attenuations cannot simply be added to get the final attenuation for any given combination of materials. The progressive loss of transmission as it passes through multiple materials is multiplicative. An algebraic computation is required to represent the final amount of radiation attenuated. Accurate attenuation of individual materials is the focus of the current study. Since each material attenuates to some degree, there will be more attenuation when both a shoe and pad are present. This is can be considered a limitation in the current study, as pads will typically only be present in conjunction with shoes. Based on the results of this study, we believe the shoes should be pulled in any case where there are concerns that lameness originates from the foot.

Solar views in conjunction with the dorsal-palmar view have proven useful in assessment of the ungular (collateral) cartilages of the foot (9, 10). The aforementioned studies revealed an excellent agreement between radiographic and scintigraphic grades of ossification of the collateral cartilages and all fractured cartilages had a greater IRU. The agreement between IRU and lesion identification on MRI was significant for the palmar process and body of the distal phalanx (7). The region of insertion of the deep digital flexor tendon (DDFT) onto the coffin bone and collateral ligaments of the distal interphalangeal joint can also be evaluated on the solar view (2, 3, 7). Current advances in Positron Emission Tomography (PET) with ^18^F-NaF in equine diagnostic imaging shows promise in providing additional and valuable information in evaluation of structures in the equine distal limb (19, 20). Although this modality utilizes similar nuclear medicine principles seen in standard equine bone scanning, the positrons are emitted at an energy of 511 keV, significantly higher than ^99m^Tc-MDP, and theoretically the attenuation would be reduced. Although the findings of this study may be applicable to this newer technique and could prevent accurate evaluation of images, PET is typically performed in conjunction with other imaging modalities (particularly computed tomography) which allow for more accurate localization of pathology and therefore require removal of the shoe.

Contrary to findings of the general musculoskeletal system, scintigraphy of the equine foot has relatively low sensitivity but is highly specific for injuries detected on MRI of the navicular bone, DDFT, and collateral ligaments of the distal interphalangeal joint (1, 2). Solar views have been used to detect abnormalities of the navicular bone by several authors (1, 2, 5, 14). There are two important considerations related to positioning of the navicular bone on the solar view that make this view extremely important (perhaps necessary) when evaluating potential pathology of the navicular bone. First, the width of the navicular bone in the transverse plane is greater than the height on the sagittal plane. Second, when the limb is positioned for a lateral view next to the gamma camera, the center of the navicular bone is at least half the width of the foot (4.4–6.6 cm) from the camera face and when positioned for the palmar views, the center of the navicular bone is much closer (1–1.5 cm) from the camera face (14). The distance from the collimator, overlying tissues and source dimensions affect the final image resolution (14, 21). This may be particularly important when evaluating horses with early or mild navicular abnormalities. In the authors experience, if a portion of the anatomy is attenuated, information about that part of the foot is lost and assessment of the image may be non-diagnostic. For example, steel shoes covering the heel and frog, will likely have a significant impact the ability to evaluate the navicular bone. This observation is quantified through this study which demonstrates that at least 50% of the radioactive emission is lost from the areas covered by the shoe and a smaller (≤10%) amount is lost through the pads. Although the pads cause significantly less attenuation, it does not seem reasonable or technically feasible to remove only the shoes and leave the pads in place.

When the horse's foot is placed directly on the camera, any material placed between the radioactive bone and the detector will absorb some amount of radiation and therefore impact the image. In the authors experience, solar images where the shoe was present and then images were repeated with the shoes removed a significant loss of anatomic detail when the shoes were present was observed.

In a clinical setting, in the authors' experience, several interesting observations have been made. Structures emitting radiation from within the center of the shoe artificially appear more intense as the uptake from the solar margins of the coffin bone is reduced, particularly if the acquisition is based on obtaining a specific number of counts rather than acquiring for a specific amount of time. This effect is due to the attenuation from the shoes. Although this may seem beneficial in specific cases (i.e., suspected navicular disease), where the shoe is eliminating extraneous radiation from other structures within the foot, if information from the surrounding structures is lost the relative importance of the findings in the center of the foot may be unknown (i.e., there may be multiple problems in the foot of which one or more are not identified). Another clinical observation seen by the author, differentiation between focal IRU in the center of the foot (navicular bone) and more diffuse IRU toward the toe (associated with the coffin bone) is not as distinguishable with shoes as without the shoes. Possible reasons for this are the area of radioactive exposure from the foot is reduced in the images with shoes present, resulting in the structures in the center of the foot generating more counts relative to the rest of the foot. This effect may be particularly evident when considering the size of the horse's foot and relative size of the shoe. In horses with smaller feet, shod with shoes with wide bars, the width between the bars of the shoes becomes smaller and the effective area of the sole that is covered by the shoe increases and consequently decreases area of the sole that is unobstructed decreases. Although not measured in the current study, the width of the shoe and width of the bars of the shoe will likely play a significant role in attenuating the clinically important radiation coming from the foot when shoes are present. Another consideration affecting the clinical image is depending on the shoe, there is an additional 1–2 cm of distance between the radioactive source and the detector. This added distance reduces image resolution. Lastly, it is important to consider horses with poor perfusion of the distal limb, resulting in increased time to acquire a diagnostic image with adequate counts (22). This increased time for image acquisition may decrease the image resolution due to inherent motion of the patient.

We are well-aware that when foot lameness is a concern, shoes may play a vital role in the health of the horse's foot. Many horses require complex orthotic and therapeutic shoeing packages where cost may be a consideration relevant to the cost of the diagnostic imaging being performed. The imaging and removal of the shoes should be performed when the farrier is capable of putting the shoes back on in a reasonable amount of time. In the author's workplace, when the shoes are removed, the foot is carefully protected with tape immediately after the scan is performed. Foam or cotton sheet pads may also be used to provide added support after the scan is complete.

The findings of this study clearly demonstrate that there is unavoidable attenuation caused by shoes and associated footwear. This leads the authors to believe there is a significant advantage to imaging the horse's bare foot on the solar view and recommend removal of the shoe prior to bone scanning of the feet. As with other imaging techniques (radiography, MRI and CT) the necessity to remove the shoes due to technical difficulties and modality requirements are often understood by the referring veterinarian and owner. This study provides scientific evidence to the equine veterinary community and clients demonstrating the attenuation of gamma radiation caused by shoes and pads and provides room for *in vivo* studies demonstrating the need and advantage of removing the shoes/pads prior to the nuclear scintigraphic imaging process.

## Data Availability Statement

The raw data supporting the conclusions of this article will be made available by the authors, without undue reservation, to any qualified researcher.

## Author Contributions

LW, MM, and NR contributed conception and design of the study. LW and MM contributed to interpretation and analysis of data. LW, MM, and BD wrote the first draft of the manuscript. LW, MM, BD, and ST participated in critical revision for intellectual content of the final manuscript. All authors read and approved the submitted version.

## Conflict of Interest

LW, MM, NR, and BD were employed by the company California Equine Orthopedics and ST is employed by Blue Ridge Equine Clinic. The authors declare that the research was conducted in the absence of any commercial or financial relationships that could be construed as a potential conflict of interest.
