# Supplementary material for: Attenuation From Shoes and Pads in Equine Nuclear Scintigraphy, With Relevance to Solar Views
Source: Front Vet Sci. 2020 Sep 24;7:516718. doi: 10.3389/fvets.2020.516718 (PMC7542681; doi:10.3389/fvets.2020.516718)
Supplement: Supplementary file 1 [file Data_Sheet_1.pdf]

Appendix 1. Images from data acquisition

Set 1 Uniformity and ROI Measurements

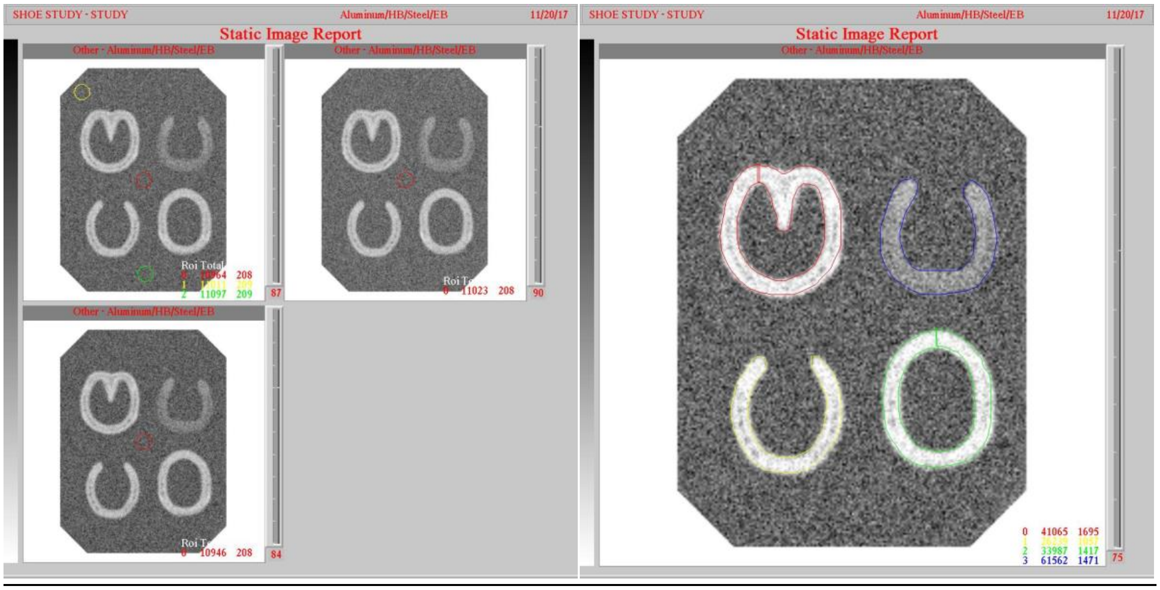

Set 2 Uniformity and ROI Measurements

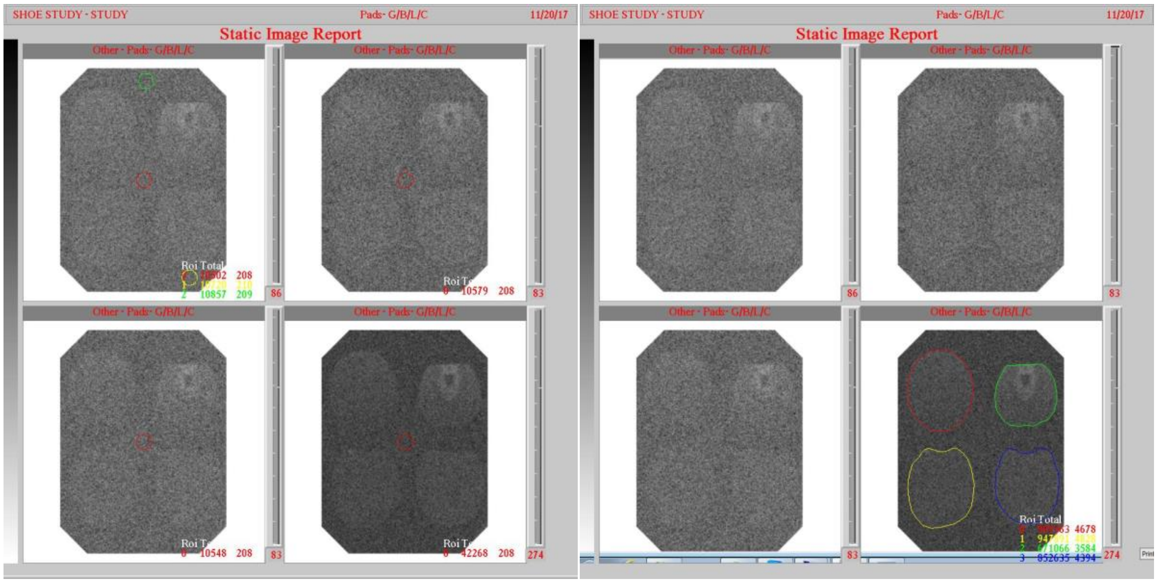

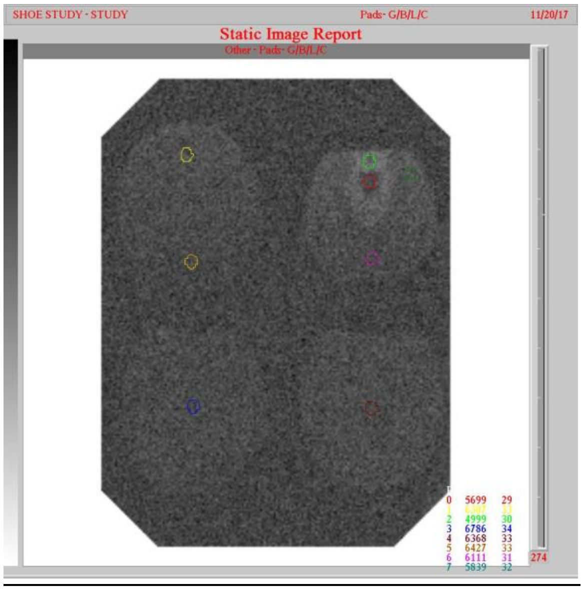

Set 3 Uniformity and ROI Measurements

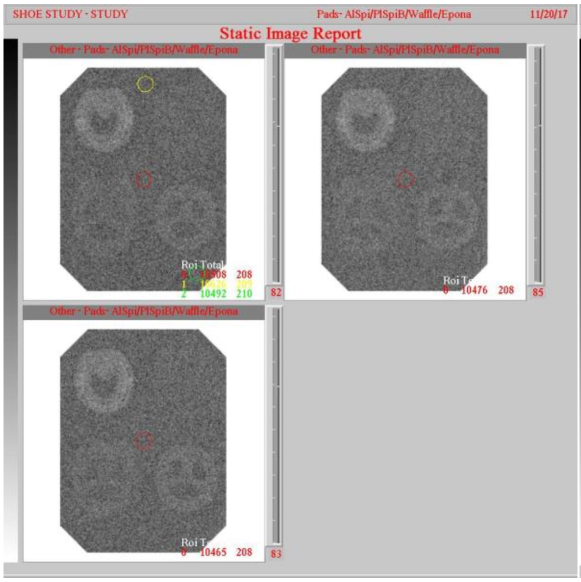

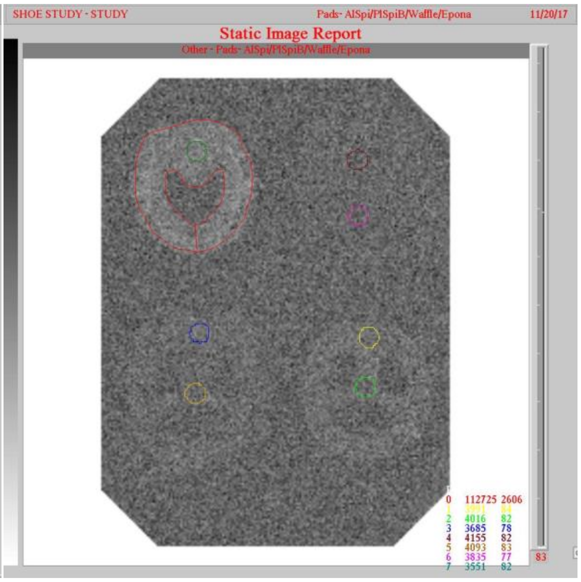

Set 4 Uniformity and ROI Measurements

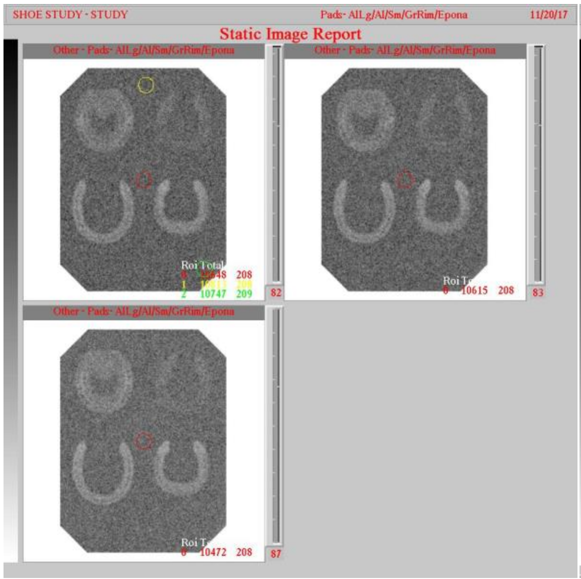
SHOE STUDY - STUDY
Pads- AlLg/Al/Sm/GrRim/Epona
11/20/17
Static Image Report
Other - Pads- AlLg/Al/Sm/GrRim/Epona
Roi Total
2 10747 209
82
0 10615 208
83
0 10472 208
87

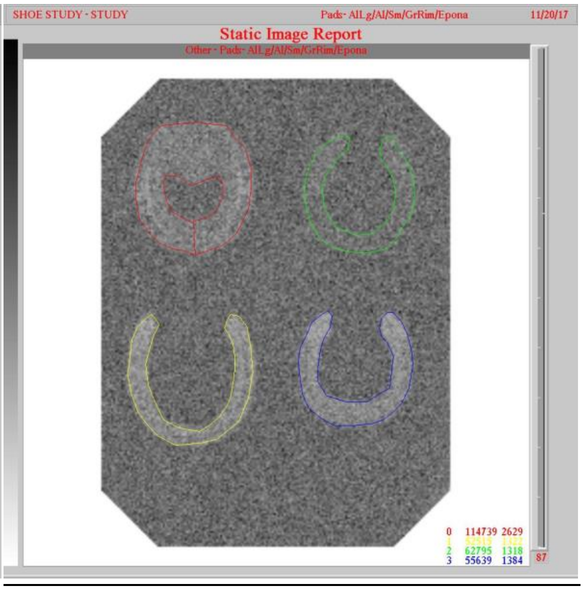
SHOE STUDY - STUDY
Pads- AlLg/Al/Sm/GrRim/Epona
11/20/17
Static Image Report
Other - Pads- AlLg/Al/Sm/GrRim/Epona
0 114739 2629
2 62795 1318
3 55639 1384
87

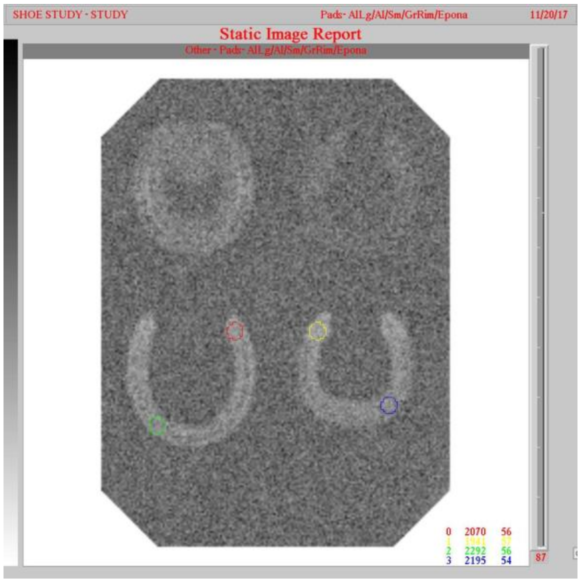
SHOE STUDY - STUDY
Pads- AlLg/Al/Sm/GrRim/Epona
11/20/17
Static Image Report
Other - Pads- AlLg/Al/Sm/GrRim/Epona
0 2070 56
2 2292 56
3 2195 54
87
